# CNN-SVM for Microvascular Morphological Type Recognition with Data Augmentation

**DOI:** 10.1007/s40846-016-0182-4

**Published:** 2016-12-10

**Authors:** Di-Xiu Xue, Rong Zhang, Hui Feng, Ya-Lei Wang

**Affiliations:** 1 Department of Electronic Engineering and Information Science, University of Science and Technology of China, Hefei, 230027 China; 2 Key Laboratory of Electromagnetic Space Information, Chinese Academy of Sciences, Hefei, 230027 China; 3 Department of Gastroenterology, The First Affiliated Hospital of Anhui Medical University, Hefei, 230022 China

**Keywords:** Microvascular type classification, Feature representation, Convolutional neural network, Support vector machine (SVM), Data augmentation

## Abstract

This paper focuses on the problem of feature extraction and the classification of microvascular morphological types to aid esophageal cancer detection. We present a patch-based system with a hybrid SVM model with data augmentation for intraepithelial papillary capillary loop recognition. A greedy patch-generating algorithm and a specialized CNN named NBI-Net are designed to extract hierarchical features from patches. We investigate a series of data augmentation techniques to progressively improve the prediction invariance of image scaling and rotation. For classifier boosting, SVM is used as an alternative to softmax to enhance generalization ability. The effectiveness of CNN feature representation ability is discussed for a set of widely used CNN models, including AlexNet, VGG-16, and GoogLeNet. Experiments are conducted on the NBI-ME dataset. The recognition rate is up to 92.74% on the patch level with data augmentation and classifier boosting. The results show that the combined CNN-SVM model beats models of traditional features with SVM as well as the original CNN with softmax. The synthesis results indicate that our system is able to assist clinical diagnosis to a certain extent.

## Introduction

Feature design for image recognition has been studied for decades. Powerful features, such as local binary pattern (LBP) [1], scale-invariant feature transform (SIFT) [2], speeded up robust features (SURF) [3], and histograms of oriented gradients (HOG) [4], have been proposed to promote the development of classical computer vision and pattern recognition tasks. However, these traditional handcrafted features are unsatisfactory for distinctive tasks, especially medical image processing.

Recently, deep learning using convolution neural networks (CNNs) has gained much success in visual recognition tasks such as image classification and object detection [5–8]. Since the descriptors acquired from these neural networks (e.g., AlexNet [5] and OverFeat [6]) are quite powerful, it is popular to treat these CNNs, trained on large natural image datasets (e.g., ImageNet [9]), as generic feature extractors. By reusing the knowledge gained from past related tasks [10–12], it is now much easier to tackle more challenging tasks such as image retrieval [5], semantic segmentation [13], fine grained recognition [14], and emotion recognition [15].

Support vector machine (SVM) is popular for classification, particularly for medical signal processing [16–18]. For recognition, great attention has been paid to the fusion of neural networks and SVM. The benefits of their combination have been confirmed by prior works on pedestrian detection [19], face recognition [20], and handwritten digit recognition [21]. Razavian et al. [14] use an off-the-shelf CNN representation with linear SVM to address recognition tasks. The results suggest it to be a strong competitor to more sophisticated and highly tuned state-of-the-art methods on various datasets.

Another way to advance recognition is called data augmentation [22], where transformations such as deformation and translation [5] have led to significant improvements in prediction accuracy and system robustness.

This paper focuses on the recognition of intraepithelial papillary capillary loops (IPCLs), a kind of esophageal microvessel, whose types are closely related to the depth of tumor invasion of esophageal squamous cell carcinoma. While the recognition results are meaningful for cancer detection and treatment [23], the task suffers from challenges such as inter-class similarity, intra-class variety, and data imbalance. Hence, efficient feature representations, strong classifiers are desired.

In this paper, we propose a CNN-SVM model for the recognition of IPCLs. The model is tested on the NBI-ME dataset (Sect. 2.2). The key idea of our method is to train a specialized CNN called NBI-Net to extract robust hierarchical features from image patches and provide them to SVM classifiers. We also investigate the feature representation ability of deep models and examine the characteristics of narrowband imaging (NBI) images through data augmentation. Comparisons with traditional feature extractors and a plain CNN model show that the proposed model outperforms them.

## Related Work

With poor prognosis when diagnosed at an advanced stage, esophageal cancer ranks as the sixth most common cause of cancer-related death [23].

NBI is a technology that enhances vessel imaging based on the spectral absorption of hemoglobin. Recent developments in narrowband imaging with magnified endoscopy (NBI-ME) and medical image processing technologies allow clear visualization of the esophageal microvascular structure, facilitating cancer detection in the early stage [24, 25].

### IPCL Type Definition

**Fig. 1:**
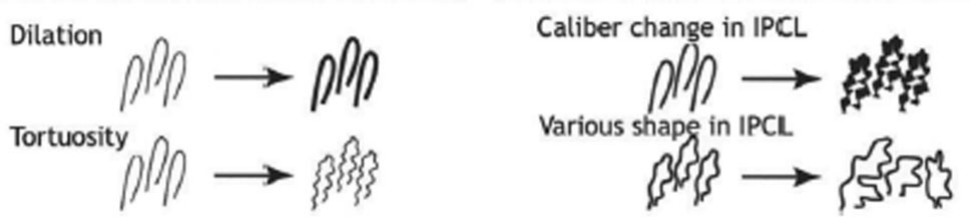
Morphological changes of vessels [23]

**Fig. 2:**
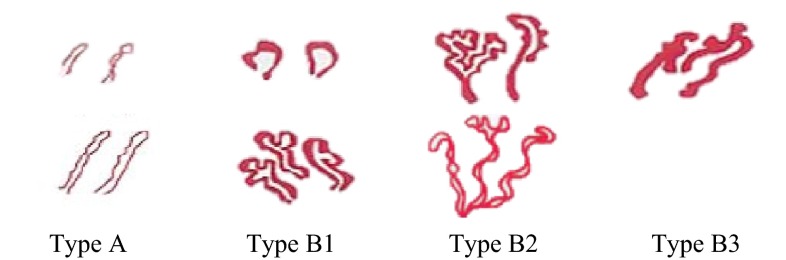
Illustration of typical IPCL types [26]

In clinical practice, IPCLs are observed as brown loops on NBI-ME images. Their types demonstrate characteristic morphological changes (Fig. 1) according to the cancer infiltration. The microvessel types are classified into four classes according to the magnified endoscopy diagnostic criteria for esophageal cancer proposed by the Japan Esophageal Society [26] and researchers [24]. The types, illustrated in Fig. 2, are defined as follows:

- *Type A* normal vessels, or vessels with slight dilation and tortuosity
- *Type B1* dilated and tortuous vessels of various diameters and shapes and with intact loop formation
- *Type B2* irregularly and dendritically branched vessels with no loop formation
- *Type B3* obviously thicker vessels than surrounding ones

### Image Acquisition and Annotation

Our NBI-ME dataset contains 261 full-size images of 67 patient cases captured from January 2013 to February 2015 at the Department of Gastroenterology, the First Affiliated Hospital of Anhui Medical University. Confirmed by biopsy of esophagectomy specimens, image regions were manually annotated after collection. Besides giving a scalar label type, label curves were carefully drawn on each original full-size image.

### Task Challenges

Data problems are sometimes the bottleneck in a pattern recognition system. For NBI image acquisition, non-uniform illumination and camera noise result in a reduction of image quality. In addition, the magnification of NBI images changes with the distance from the tissue to the camera lens. Thus, parts of raw images must be discarded due to image distortion.

**Fig. 3:**
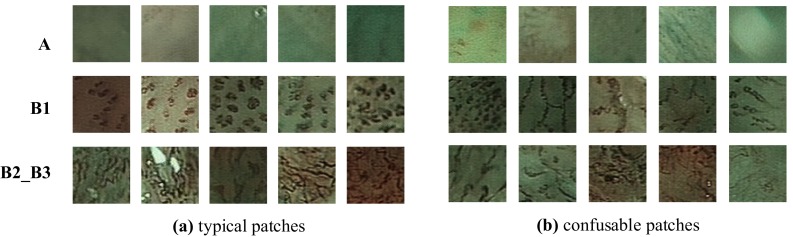
**a** Typical and **b** confusable patches

The classification task suffers from inter-class similarity and intra-class variety (Fig. 3). Medically, tumors progress gradually and continuously from low to high grade. However, IPCLs are factitiously classified into four discrete types so that it is sometimes difficult to distinguish two adjacent types. The texture pattern of IPCLs varies from case to case, leading to highly intra-class variety.

In addition, data imbalance is a problem for model optimization. For instance, a class with fewer training samples is easily belittled when the optimal target is minimizing the training error of the whole dataset. As lesions for B2 and B3 are much fewer than those for A and B1, to mitigate data imbalance, we simplified the problem to a three-class (A, B1, and B2_B3) classification task, on condition that types B2 and B3 have much in common.

### Conventional Features

According to clinical experience, texture and shape play crucial roles in microvascular morphological type recognition. Conventional features, namely pyramid histogram of words (PHOW) [27], LBP, and pyramid histogram of oriented gradient (PHOG), are used in our experiments.

PHOW, an effective texture feature, is a combination of SIFT and the bag of words model. Variants of dense SIFT descriptors, extracted at multiple scales, are clustered into visual words and the histograms of these words are treated as descriptions of images. PHOG is similar but describes shape. LBP is a set of local descriptors that capture the appearance of an image cell (a small neighborhood around a pixel), recording local pixel intensity difference.

### Support Vector Machine

SVM was originally proposed for binary classification. Supposing a training set $S = \{ x_{i} ,y_{i} \}$, where feature vector $x_{i} \in R^{d}$ and label scalar $y_{i} \in \{ - 1,1\}$, the soft margin SVM tries to find a hyperplane that satisfies the following constrained optimization:

1 $$arg\mathop {min}\limits_{{{\mathbf{w}},\xi ,b}} \quad \frac{1}{2}{\mathbf{w}}^{T} * {\mathbf{w}} + C\sum\limits_{i = 1}^{n} \,\xi_{i}$$

2 $${\text{subject}}{\kern 1pt} {\kern 1pt} {\text{to}}{:}\left\{ {\begin{array}{*{20}c} {y_{i} ({\mathbf{w}}^{T} * x_{i} + b) \ge 1 - \xi_{i} } \\ {\xi_{i} \ge 0,{\kern 1pt} {\kern 1pt} i = 1,2, \ldots ,m} \\ \end{array} } \right.$$

where ${\mathbf{w}}$ is the weight vector for $x$, $b$ is the intercept of the hyperplane, vector $\xi$ contains the slack variables, and $C$ is the adjustable penalty parameter controlling the trade-off between the maximization of the margin and the minimization of the classification error.

By importing a kernel function, SVM is able to solve a nonlinear separable problem by transforming the feature vector into a high-dimensional space.

## Architecture

We want to design a data-adaptive and customer-friendly system to aid clinical diagnosis. For real-time recognition, a batch processing program is required to train and test images parallelly at a constrained time cost. Advanced CNNs, sped up by a GPU, are an excellent match for this job.

**Fig. 4:**
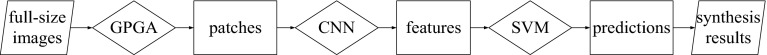
Flow diagram of proposed system

The flow diagram of our system is shown in Fig. 4. In our system, image patches are generated from marked regions for the CNN by a greedy patch-generating algorithm (GPGA) and the locations of all patches are recorded for further synthesis via Gaussian-weighted voting. The CNN functions as a trainable feature extractor and the SVM acts as a predictor.

### Greedy Patch-Generating Algorithm

**Fig. 5:**
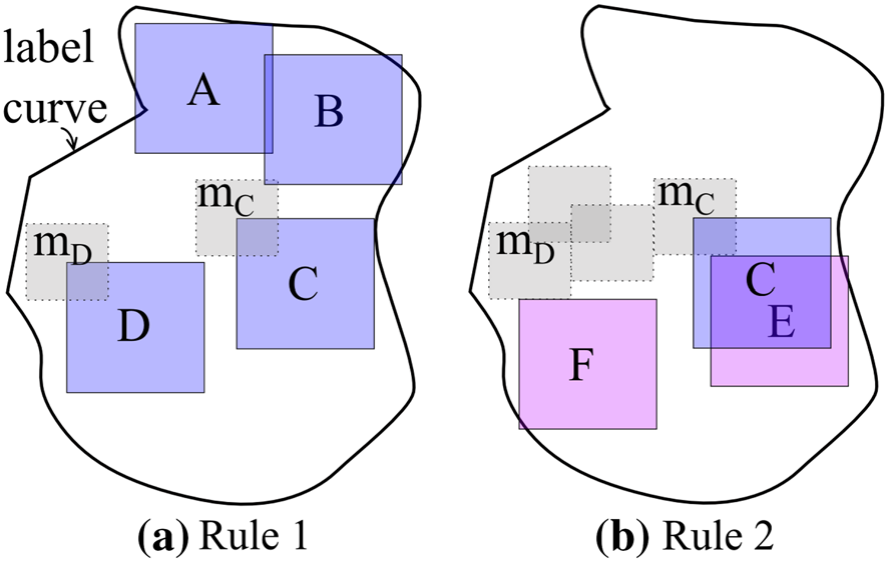
Schematic of greedy patch-generating algorithm
- Rule 2, the area-overlapping-percentage of any two patches should be exempted from too high (≤75%), e.g., patches *C* and *E* in Fig. 5b.

Since fixed-size image patches are required by the CNN, we employ a GPGA to take advantage of marked regions following these two rules:

- Rule 1, most (≥95%) of a patch must be bounded within the label curve, e.g., patches *A* and *B* in Fig. 5a

Rule 2 can be achieved by invalidating a smaller gray area (e.g., *m*_*C*_ for patch *C*, *m*_*D*_ for patch *D*) around the upper-left point of previously generated patches. These gray areas are then marked unreachable for new patches.

### Convolutional Neural Network

We build our network following the popular three-stage designs (i.e., convolutional layers, pooling layers, and fully connected layers) with modifications, including rectified linear units (ReLU), local response normalization (LRN), and dropout, to prevent overfitting.

The input layer size is one of the most important parameters when building a CNN. For example, the typical 5-layer LeNet-5 [28] handles 28 × 28 images and the 8-layer AlexNet takes images of size 256 × 256. A deeper and wider network is expected to learn richer hierarchical features, but needs a larger number of training samples and more iterations for fine-tuning. Limited by the amount of data,^1^ it is difficult to train and tune a large network.

**Fig. 6:**
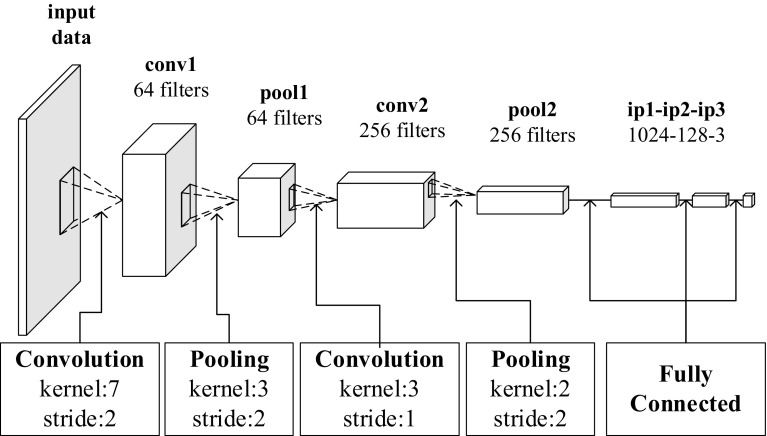
Architecture of proposed NBI-Net

By taking account of our patch size and sample magnitude (see Sect. 4 for details), we started from a five-layer model. Inspired by AlexNet and GoogLeNet [8], we increased the kernel size of the first convolutional layer (conv1) from 5 to 7 to widen the filter “sight” and placed a max pooling layer (pool1) with a size of $z \times z$ = $3 \times 3$ and stride $s$ = 2. A larger kernel also could speed up training and decrease CNN depth. We set $s < z$ and obtained an overlapping pooling layer to reduce overfitting. Smaller convolutional and pooling kernel sizes were chosen, as features of higher abstraction would be captured by the second convolutional layer (conv2). The last three layers were 1024-d, 128-d, and 3-d fc layers (fc1 to fc3), with softmax as the output function. The width of each layer was tuned according to the system demand analysis. The architecture of our CNN and the blob shape before fc layers are summarized in Fig. 6 and Table 1.

**Table 1:** Blob shapes of proposed NBI-Net model

	Input data	conv1	pool1	conv2	pool2
Default	3 × 64 × 64	64 × 29 × 29	64 × 14 × 14	256 × 12 × 12	256 × 6 × 6
Cropped	3 × 56 × 56	64 × 25 × 25	64 × 12 × 12	256 × 10 × 10	256 × 5 × 5

Layers conv1, conv2, ip1, and ip2 were equipped with ReLU [29] to avoid gradient vanishing and speed up convergence. In addition, we used dropout in the fully connected layers, with a dropout probability of 0.5, to help prevent units from co-adapting and generate more robust features by learning on different random subsets [5, 30].

Our system was built with Caffe [31], a powerful deep learning framework developed by the Berkeley Vision and Learning Center (BVLC), with the NVIDIA CUDA cuDNN [32] library and trained on a single NVIDIA GPU.

## Experiments

We selected a patch width of 64 as a trade-off between accuracy and data proportion. About 6.5 k patch samples were generated using GPGA. The performance was measured using average precision (AP) on the patch level.

In addition to CNN, we implemented extractors of PHOW, LBP, and PHOG. Raw RGB patches were directly fed to each feature extractor. No pre-processing method was applied except mean component removal in CNN.

When applying the cross-validation strategy for testing model performance, we sliced the dataset on the case level rather than the patch level because images from the same patient case were similar in texture pattern and patches were generated with an overlap.^2^ The classification accuracy in table was the aggregated accuracy of all folds.

The time cost depended on the dataset and model complexity, and could be sensitive to programming and hardware. In this work, we trained our models on NVIDIA GPUs (GTX 980 and GTX 970). During the CNN training stage, we used an initial learning rate of 0.01 or 0.005 and scheduled two tenfold decreases for fine-tuning. The models were iterated with 50 epochs (about 30,000 gradient steps). Most models finished in several minutes, but the largest one took about 2 h due to data augmentation.

A GPU-accelerated SVM [33, 34] has been suggested to deal with high-dimensional feature vectors. Principal component analysis (PCA) is optional and should be carefully used for dimensionality reduction.

In Sect. 4.1, we examine the characteristics and distribution of the NBI dataset and improve the precision of NBI-Net via data augmentation. The CNN representation ability analysis and performance comparison of models are respectively presented in Sects. 4.2 and 4.3.

### Data Augmentation

To be robust, a model for pattern recognition should make predictions that are invariant to various inputs of a given label. A straightforward approach is to collect a large number of training samples with abundant variation, regardless of the difficulties in data collection and labeling.

Another way to deal with this problem is data augmentation, which is achieved by adding sample replicas with label preservation. Various kinds of affine transform may take effect depending on the characteristics and distribution of a dataset. We apply rescaling, rotation, and flipping on the full-size images and used Caffe embedded cropping for patches. We prioritized operation on the image level over that on the patch level because operation on the image level could afford more randomness of data augmentation benefited from our GPGA.

#### Rescaling

**Fig. 7:**
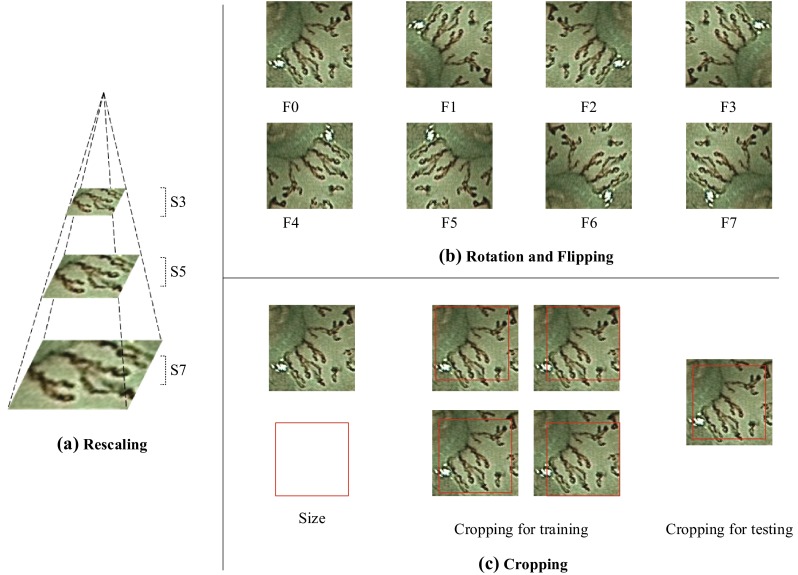
Illustration of data augmentation with **a** rescaling, **b** rotation and flipping, and **c** cropping

The NBI images were acquired on different scale factors. The non-normalized scale factor may confuse our model and weaken generalization ability. For rescaling, as shown in Fig. 7a, images were rescaled on a spatial pyramid using:

3 $$\frac{{d_{i} }}{{d_{j} }} = k^{i - j}$$

where $d_{i}$ is the length of the side for mode $S_{i}$. The factor $k$ was set to $\sqrt 2$ from experience.

#### Rotation and Flipping

Unlike natural images (e.g., images of human faces or buildings), there is no clear principal direction of NBI images. Rotation and flipping were introduced to strengthen rotational invariance. In this work, the dataset was augmented via roughly rotating each image by every 90 degree and doubled by flipping. This produced eight modes, as shown in Fig. 7b.

#### Cropping

Cropping was implemented by randomly shifting on both horizontal and vertical lines in the training stage. For testing, patches were cropped at the center, as shown in Fig. 7c. The length proportion of a cropping was 56/64 for NBI-Net, 227/256 for AlexNet, and 224/265 for VGG-16 or GoogLeNet.

Table 2 shows the configurations and relations of models A-I. Model A, NBI-Net without any data augmentation, acted as the baseline in this test. The steady increase in the recognition rates of models A, G, and H shows that rotation and flipping operations were effective. Unlike rotation and flipping, which have high priority, the scaling operations had to be picked carefully, as models A-F show. While the robustness of CNN was improved with rescaling modes S4 and S6, modes S3 and S7 seem to introduce a lot of noise, which confused models E and F, resulting in a decrease in accuracy. The intensity of the rescaling should be gradually increased. For all NBI-Net models, cropping did not improve accuracy, as it did for models trained on ImageNet, such as AlexNet (see Table 3). Although cropping reduces precision, it may prevent overfitting in the visualization of model losses.

**Table 2:** Configurations of datasets under augmentation and CNN average precision

NBI-Net model	Rescaling mode	Rotation/flipping mode	Data complexity	AP w/o cropping	Compared to base	AP w/cropping	Compared to base
	S5	F0	1	90.03	(Base)	89.42	−0.61
B	S4–S5	F0	×1.3	90.28	0.25	89.63	−0.40
C	S5–S6	F0	×3.6	91.45	1.42	89.45	−0.58
D	S4–S6	F0	×3.9	*91.87*	*1.84*	90.62	0.59
E	S4–S7	F0	×10.2	90.43	0.40	88.69	−1.34
F	S3–S6	F0	×3.9	91.65	1.62	90.13	0.10
G	S5	[F0–F7]/2	×4	91.42	1.39	90.11	0.08
H	S5	F0–F7	×8	*91.80*	1.77	90.00	−0.03
I	S4–S6	F0–F7	×3.9 × 8	***92.31***	***2.28***	91.24	1.21

“[]/2” represents a random 50% downsampling

The top results have been styled with bold and italic

The best results of comparative group are styled with italic

**Table 3:** Average precision of CNN models with linear SVMs

Model	Feature layer			Avg
	fc1	fc2	fc3	
AlexNet	87.14	88.48	87.29	87.64
AlexNet Cropping	87.91	88.93	87.99	88.28
VGG-16	89.20	90.02	89.65	89.62
VGG-16 Cropping	89.88	91.67	90.68	*90.74*
GoogLeNet	85.62	86.20	–	85.91
GoogLeNet Cropping	87.57	86.83	–	87.20
NBI-Net (model A)	90.93	90.64	91.21	90.93
NBI-Net Aug (model I)	92.87	92.38	92.97	***92.74***

Layer “fc1” is first fully connected layer before softmax or layer before softmax1 in GoogLeNet, or named “fc6” in AlexNet

Principal component analysis (PCA) here is optional and should be carefully used for dimensionality reduction

The top results have been styled with bold and italic

The best results of comparative group are styled with italic

Model I without cropping (NBI-Net Aug), which is a combination of models D and H, was best, improving accuracy by 2.28% against the baseline.

### CNN Feature Descriptor Analysis

The ImageNet Large Scale Visual Recognition Challenge (ILSVRC) has become the standard benchmark for object recognition. It contains millions of images belonging to thousands of object categories. State-of-the-art models AlexNet, VGG-16, and GoogLeNet are all top winners of this challenge. While models trained on ImageNet can be transferred well to natural image sets such as Caltech-101, there is currently no clear understanding of how they will do on medical image sets.

The ImageNet dataset consists of thousands of object classes, with only a small number resembling an NBI scene. A comparison was made between pre-trained models and our NBI-Net trained on the NBI image set. For testing, NBI patches were fed to CNN feature extractors. Since features from fully connected layers are more discriminative than those from convolutional layers [12], linear SVMs were applied after fully connected layers.

According to Table 3, all CNN models have a recognition rate of at least 85%. This indicates that stacked convolutional networks show obvious adaptability to feature descriptions.

The accuracy of VGG-16 Cropping, the best performing generic model trained on ImageNet, is close to that of NBI-Net trained on the NBI dataset. The results imply a similarity between natural and medical images in terms of basic feature representation. Starting from a pre-trained model for IPCL recognition is thus worthy of consideration.

### Model Comparison

Here, we only compare models; feature fusion and model ensembles are beyond the scope of this study.

Some key parameters of the experiments are as follows. The PHOW feature was extracted from the 2-level spatial pyramid $\{ 4 \times 4,2 \times 2\}$ with 200 visual words. The PHOG feature was obtained from the 4-level pyramid $\{ 8 \times 8,4 \times 4,2 \times 2,1 \times 1\}$ with 8 angle bins. Uniform-58 LBP was applied to each cell of size $16 \times 16$. For the CNN, we took the output of fully connected layers ip1, ip2, and ip3. Both linear SVM and SVM with a radial basis function (RBF) kernel were used in the experiments except for the original CNN (CNN-softmax group). For multiclass problems, we adopted the one-against-one strategy for SVMs. The class that received the most votes won.

In Table 4, the results show that CNN models with linear SVM are equivalent to CNN models with SVM with the RBF kernel, which means that CNN features are almost linearly separable whereas conventional features are not. Linear SVM was used for further study in consideration of algorithm complexity.

**Table 4:** Average precision of models using SVM and dimension of features

Feature extractor	Average precision	Average precision	Feature dimension
	(Linear SVM)	(SVM with RBF kernel)	
PHOW	80.24	*85.16*	4000
LBP	82.39	82.90	928
PHOG	49.04	62.60	680
AlexNet Cropping	88.28	88.12	4096/4096/3
VGG-16 Cropping	90.74	90.49	4096/4096/3
GoogLeNet Cropping	87.20	87.33	1000/1000
NBI-Net Aug	***92.74***	92.70	1024/128/3

The top results have been styled with bold and italic

The best results of comparative group are styled with italic

**Fig. 8:**
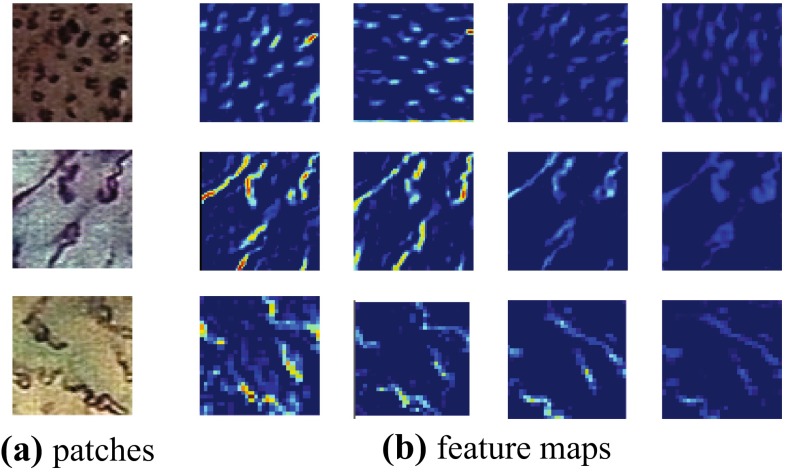
**a** Patches and **b** their feature maps from conv-1 layer of NBI-Net

A comparison among rows shows that CNN features significantly outperform conventional handcrafted features. It can also be verified from the visualization of middle-layer feature maps (shown in Fig. 8) that CNN features fit the “disorganized” IPCL pattern very well, which is difficult to achieve using manual design.

A comparison between hybrid CNN-SVM (NBI-Net with linear SVM) and plain CNN (NBI-Net with softmax) is shown in Table 5. The fusion of CNN and SVM slightly boosted accuracy by 0.90% (0.43% with Aug). The gain is mainly due to the use of a different optimization criterion. The learning algorithm of softmax is based on empirical risk minimization, which attempts to minimize the prediction loss on the training set. In contrast, SVM aims to minimize the generalization error by using structural risk minimization principles for the testing set. As a result of a maximized margin, the generalization ability of SVM is greater than that of softmax.

**Table 5:** Accuracy of NBI-Net models with softmax and linear SVM

Model	Softmax	Linear SVM	Classifier boosting
NBI-Net	90.03	90.93	0.90
NBI-Net Aug	92.31	***92.74***	0.43

CNN = NBI-Net with softmax

CNN-SVM = NBI-Net with linear SVM

Aug (Augmentation) is optional for both CNN and CNN-SVM

The top results have been styled with bold and italic

**Fig. 9:**
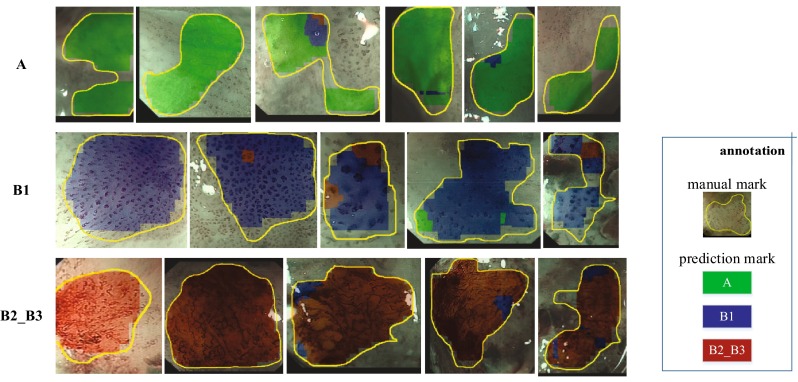
Synthesis results for full-size images. Representative images of labels **A**, **B1**, and **B2_B3** are placed in first, second, and third rows, respectively. Recognized IPCL regions are colored in *green*/*blue*/*red* for types **A**/**B1**/**B2_B3**

A combination of data augmentation and classifier boosting improved accuracy by 2.71% (=92.74% − 90.03%) and led to a high recognition rate of 92.74%. Figure 9 shows the synthesis results and the original mark curves; our system offers correct prediction in most regions. Thus, our system may be able to assist clinical judgement to a certain extent.

## Conclusion and Future Work

In this paper, a patch-based system with a hybrid CNN-SVM model was proposed for IPCL recognition to aid clinical diagnosis. The performance of the CNN model was improved by data augmentation and classifier boosting. Experimental results show that features learned by the CNN beat manually designed features in terms of efficiency and linear separability. A switch from softmax to SVM appears to be beneficial for generalization ability.

For future work, the IPCL recognition precision will be further increased by using a larger dataset, better understanding of IPCLs types, and the fine-tuning of pre-trained CNN models.

## Footnotes

1 Although a data augmentation can help increase the number of patches, the data distribution is still limited.

2 It may be cheating to distribute patches from the same case or the same full-size image to the training set and testing set concurrently.
